# Comparing fractional CO₂ laser and needling-based modalities in facial acne scar treatment: a comprehensive systematic review and meta-analysis

**DOI:** 10.1007/s10103-026-04905-5

**Published:** 2026-06-23

**Authors:** Asia Batool, Muhammad Sharjeel Abbas, Fakhar e Mahin, Javeria Nawaz, Syeda Masooma Jafri, Alisha Ahmed, Huzaifa Sabir Nawaz, Tayyaba Ikram Qazi, Muhammad Salman Khalid, Syeda Maham Guftar Shah, S. M. Aleem Hussain, Meer Murtaza, Muhammad Talha, Hasibullah Aminpoor

**Affiliations:** 1 Sir Syed College Of Medical Sciences, Karachi, Pakistan; 2 Gomal Medical College, D. I. Khan, Pakistan; 3 Fatima Jinnah Medical University, Lahore, Pakistan; 4 Dow University of Health Sciences, Karachi, Pakistan; 5 Jinnah Sindh Medical University, Karachi, Pakistan; 6 Services Institute of Medical Sciences, Lahore, Pakistan; 7 Khyber Medical College, Peshawar, Pakistan; 8 University of the Punjab, Lahore, Pakistan; 9 Wah Medical College, Punjab, Pakistan; 10 Liaquat University of Medical & Health Sciences, Jamshoro, Pakistan; 11 Faculty of Medicine, Kabul University of Medical Sciences “Abu Ali Ibn Sina”, Kabul, Afghanistan

**Keywords:** Acne Scars, Fractional CO₂ Laser, Microneedling, Post-inflammatory Hyperpigmentation, Randomized Controlled Trial, Meta-Analysis

## Abstract

Facial acne scarring is a widely prevalent dermatological disease manifested as a permanent aftereffect of acne vulgaris. While fractional CO₂ laser and needling procedures are essential to treating atrophic acne scars, comparative and safety data remains limited. This systematic review and meta-analysis directly compares their efficacy and safety. According to PRISMA guidelines, PubMed, Cochrane, Google Scholar, and ClinicalTrials.gov were systematically searched. Only studies comparing fractional CO₂ laser with needling-based monotherapy treating facial atrophic acne scars in adults were included. A random-effects model was employed for meta-analysis using Review Manager (RevMan) to compare efficacy and safety outcomes. A total of 17 studies were included in the meta-analysis. For primary efficacy outcomes, the results were non-significant for mean scar score reduction (SMD: -1.08, 95% CI: [-2.48, 0.32], I² = 97%) or mean quantitative scar reduction (SMD: 0.12, 95% CI: [-0.80, 1.04], I² = 92%), with significant heterogeneity. However, categorical treatment success was significantly high for CO₂ laser (RR: 1.10, 95% CI: [1.01, 1.20], I² = 0%). For safety outcomes, fractional CO₂ laser showed significantly high risk of post-inflammatory hyperpigmentation (PIH) as compared to standard microneedling (RR: 3.04, 95% CI: [1.49, 6.21], I² = 3%), but showed significantly low pain scores as compared to RF microneedling (SMD: -0.74, 95% CI: [-1.27, -0.21], I² = 0%). No significant differences were found for erythema, crusting/scabbing, or edema. Both fractional CO₂ laser and microneedling procedures show comparable efficacy in treating atrophic acne scars, however, patient variables such as skin type, scar type, and possible complications should be taken into consideration. Moreover, further tailored therapies are crucial to treating acne scars with low risk for complications.

**Supplementary information:**

The online version contains supplementary material available at 10.1007/s10103-026-04905-5.

## Introduction

Facial acne scarring is a common, often permanent sequela of acne vulgaris that imposes substantial individual and societal burden. Acne affects an estimated 9.4% of the global population and remains one of the most prevalent dermatologic diseases worldwide [1]. Global burden analyses estimate hundreds of millions of prevalent cases and rising age-standardized rates in many regions, emphasizing acne’s continuing public-health relevance and the downstream problem of post-inflammatory scarring [2, 3]. Atrophic facial acne scars in particular are strongly associated with impaired quality of life, social avoidance, anxiety, and depressive symptoms, and measurable reductions in well-being across diverse populations—these humanistic and economic consequences make effective, evidence-based scar therapies a priority for dermatologic research and practice [4, 5].

Acne scars are heterogeneous—typically classified as ice-pick, boxcar, and rolling atrophic types—which reflect divergent dermal injury and remodeling pathways [6]. Validated clinical grading instruments (Goodman & Baron qualitative and quantitative scales and the Echelle d’Evaluation Clinique des Cicatrices d’Acne [ECCA]) and objective volumetric imaging methods exist but are used inconsistently across trials [6–8]. Pathophysiological models implicate inflammation, altered wound healing, matrix degradation, and defective collagen deposition; these mechanisms explain why modalities that stimulate controlled dermal remodeling are central to contemporary management strategies [9].

Ablative fractional carbon dioxide (CO₂) laser resurfacing and needling-based approaches (mechanical microneedling and radiofrequency microneedling) dominate procedural treatment for atrophic acne scars because both promote neocollagenesis and tissue remodeling through different injury patterns and depths [10–12]. Systematic reviews and meta-analyses support the efficacy of microneedling (as monotherapy and in combination with biologics such as platelet-rich plasma) and of ultra-pulse/ablative fractional CO₂ lasers for depressed scars, but effect estimates vary and many primary studies are small, single-center, or evaluate combination therapies rather than direct monotherapy comparisons ([10, 11, 13, 14]). Several studies report substantial improvements with both modalities, yet outcomes vary by scar type, outcome metric, and follow-up interval [15, 16].

Importantly, comparative effectiveness and safety data remain limited. Recent trials and split-face studies show comparable improvement between fractional CO₂ laser and microneedling modalities for many patients, but differences in downtime, pain, and adverse events—notably post-inflammatory hyperpigmentation (PIH) in darker skin—complicate interpretation and patient selection [17–22]. Heterogeneity in protocols, inconsistent use of validated scar scores, short follow-up durations, and sparse head-to-head randomized evidence produce uncertainty about which monotherapy best balances efficacy and safety across scar subtypes and skin phototypes [11, 14, 17–22].

To address these gaps, we conducted a systematic review and meta-analysis comparing fractional CO₂ laser monotherapy with needling-based monotherapies for facial atrophic acne scars. The review aims to quantify comparative efficacy, evaluate safety outcomes (including PIH), and explore effect modification by scar type and skin phototype to inform evidence-based clinical decision-making.

## Methods

### Study proposal

This systematic review and meta-analysis was conducted in accordance with the PRISMA (Preferred Reporting Items for Systematic Reviews and Meta-analysis) 2020 statement and methodological guidance for systematic reviews of prognostic factor studies [23]. The study protocol was retrospectively registered on PROSPERO (International Prospective Register of Systematic Reviews) (PROSPERO Record ID CRD420251240708) in November 2025, with no deviations in study objectives and analysis from the registered protocol.

### Eligibility criteria

The following PICO (Population, Intervention or exposure, Comparison and Outcome) elements was used as inclusion criteria for the systemic review and meta-analysis: [1] Population : Adults with (facial) acne scars (any type) [2] Intervention : Fractional CO₂ laser monotherapy [3] Comparison: Microneedling monotherapy/RF microneedling/Dermaroller [4] Outcomes : Mean percentage reduction in acne scar severity score from baseline, Mean quantitative scar score reduction (mean difference) using validated grading systems, Categorical treatment success (≥ 50% reduction or ≥ 2-grade improvement in scar severity), Mean pain score measured by Visual Analogue Scale (VAS), Incidence of post-inflammatory hyperpigmentation (PIH), Incidence of post-procedure erythema, Duration of erythema/downtime, Incidence of crusting/scabbing, Incidence of edema, other reported complications and adverse effects if any.

**Fig. 1:**
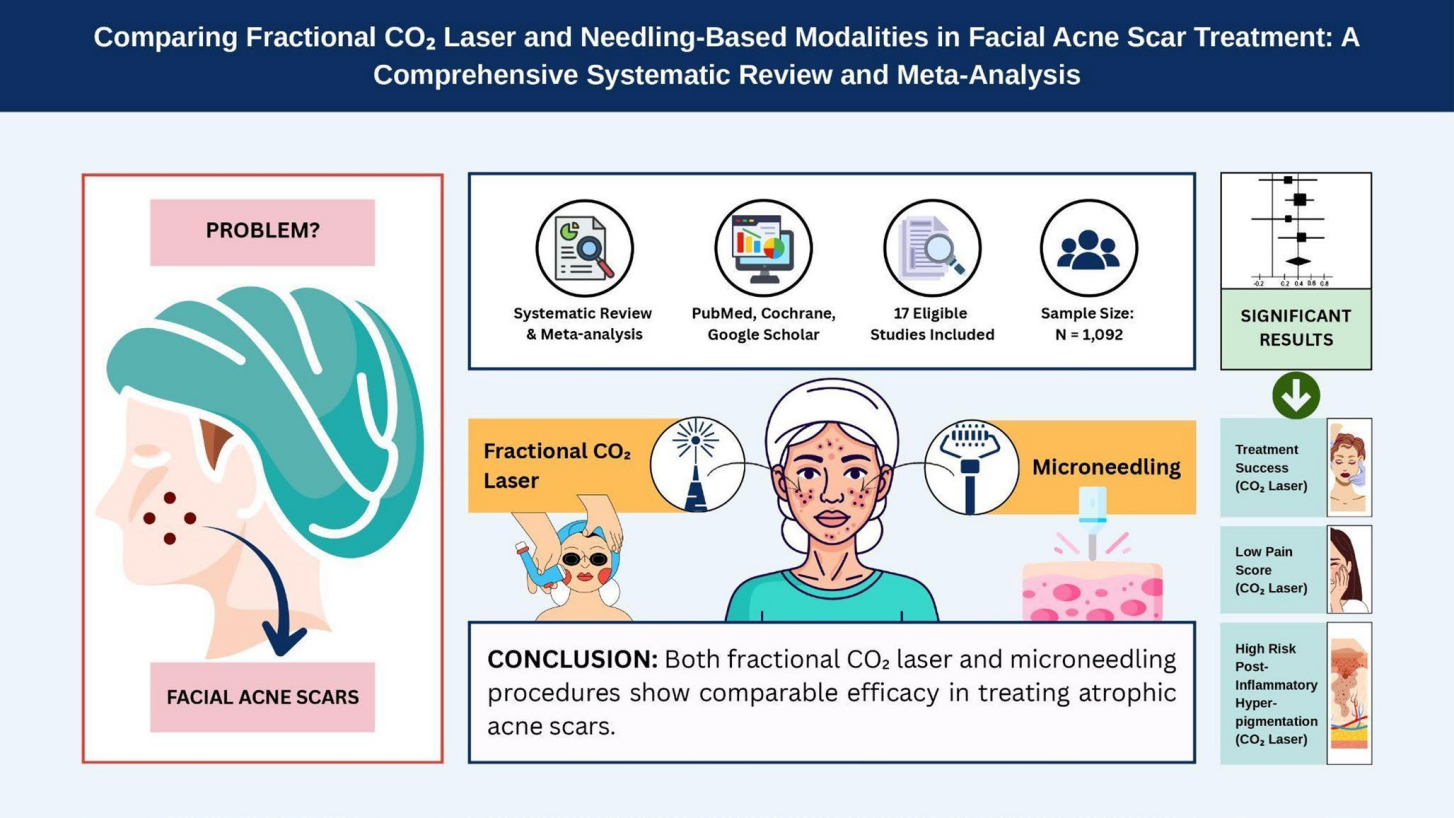
Graphical abstract of systematic review and meta-analysis comparing Fractional CO₂ Laser and Needling-Based Modalities in Facial Acne Scar Treatment

Studies with populations other than the adult population (ages 18 years or older), single arm studies, non-extractable data, non-English Publications, or animal studies were excluded (Fig. 1).

### Literature review and search strategy

**Fig. 2:**
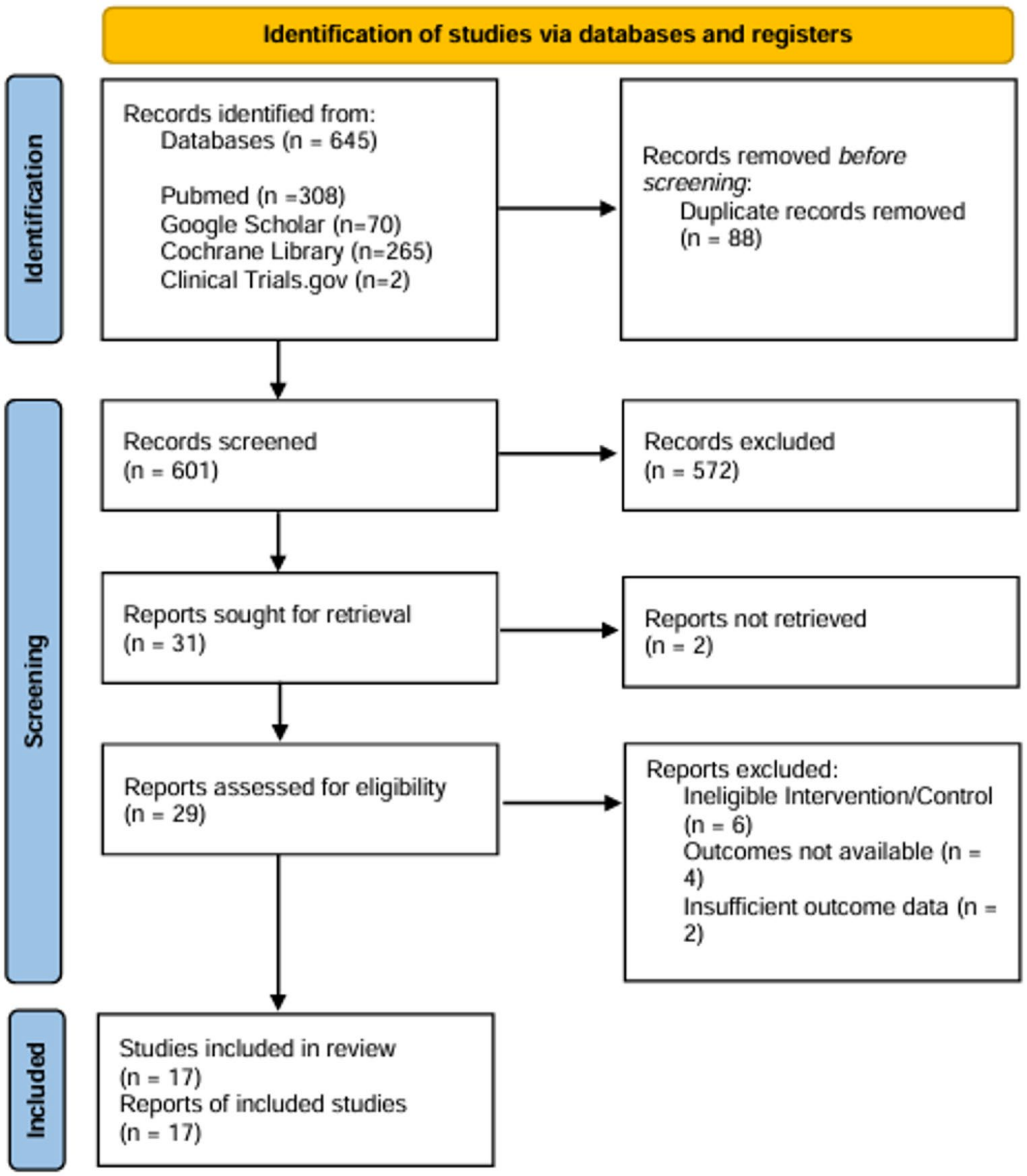
PRISMA flow diagram

A thorough literature review was done by two independent reviewers on following search engines: Pubmed, Cochrane, Google scholar and Clinical trials. The search strategy for Pubmed was: “Microneedling“[Mesh] OR microneedling[tiab] OR “radiofrequency microneedling“[tiab] OR (“Microneedling“[Mesh] AND (“Radiofrequency Ablation“[Mesh] OR radiofrequency[tiab])) OR “Carbon Dioxide Lasers“[Mesh] OR (fractional[tiab] AND “Carbon Dioxide Lasers“[Mesh]) OR “fractional CO2 laser“[tiab] OR “CO2 laser“[tiab]) AND ((“Cicatrix“[Mesh] AND “Acne Vulgaris“[Mesh]) OR “acne scars“[tiab] OR (“atrophic“[tiab] AND (“acne scars“[tiab] OR “Cicatrix“[Mesh]))). Studies from inception till September 2025 were sought and all the data from search engines was uploaded on Rayyan. A total of 573 articles were found in database search: 308 from PubMed and 265 from Cochrane. After the removal of 88 duplicates, 485 records were subjected to title and abstract screening. Of these, 49 articles further underwent full text assessment for eligibility, and 17 studies met the inclusion criteria which were included for the qualitative and quantitative analysis. This is presented in Fig. 2.

### Data extraction

Data extraction was done for the included studies in a standardized manner by two independent reviewers independently on an Excel sheet. The data was extracted and recorded in the following order from each study: [1] Title [2] Year of Publication [3] Author [4] Journal [5] Study Design [6] Baseline Characteristics [7] Type of Acne scar [8] Goodman and Baron’s Quantitative Assessment [9] Skin Phenotype [10] Baseline Scar score [11] Scar Duration [12] Mean % reduction in Scar Score [13] Mean Pain Score (VAS) [14] Post- treatment inflammatory hyperpigmentation (PIH) [15] Erythema incidence and duration [16] Other Side effects if any.

For all characteristics, dichotomous data was displayed as a number and percentage and continuous variables were displayed as mean difference (MD). For dichotomous data, risk ratios (RRs) were utilised with a p-value of < 0.05 and 95% Confidence Interval (CI) considered to be statistically significant.

### Statistical analysis

Comparative data was represented utilising forest plots by using Review Manager Version 5.4 [24]. Meta-analysis of data utilised a random effects model to account for the likely differences in effect estimates between studies due to the pre-empted heterogeneity between these studies and their techniques. Heterogeneity was assessed utilising I2, Chi2 statistics and Tau2 statistics with a P value of < 0.05 used to assess the likelihood of heterogeneity. An I2 statistic of 0–40% suggests heterogeneity is unlikely important, 41–50% may represent moderate heterogeneity, 51–74% representing severe heterogeneity and 75–100% representing considerable heterogeneity. A random effects model was utilised given the likely heterogeneity between techniques and institutions. Publication bias was assessed utilising a funnel plot and Egger’s test in the instance of more than ten papers being analysed.

### Sensitivity analysis

We performed leave-one-out sensitivity analyses for Mean Reduction in Acne Scar Score, Mean Quantitative Scar Reduction, Post-Procedure Erythema, Edema, to assess the effects of influential studies on the pooled analysis. Studies were sequentially removed, and the data were reanalyzed to ensure the stability of the pooled effects.

### Endpoints

Outcomes to be analyzed included: Mean Reduction in Acne Scar Score, Mean Quantitative Scar Reduction, Categorical Treatment Success, Pain Score (VAS), Post-Inflammatory Hyperpigmentation (PIH), Post-Procedure Erythema, Crusting/Scabbing, and Edema.

### Quality assessment

Two independent reviewers performed risk of bias assessment independently by using the Cochrane ROBINS-I assessment tool [25] for non-randomized studies and ROB-2 by Cochrane Review of Studies [26] for randomized studies and resolved the conflicts by mutual discussion. The studies were graded as low, moderate or high risk of bias.

### Certainty of evidence

The certainty of evidence for the primary outcomes and secondary outcomes was assessed using the Grading of Recommendations Assessment, Development and Evaluation (GRADE) approach [27].The included studies were observational and randomised controlled trials, the certainty of evidence was initially rated as low. The certainty was then evaluated across the following domains: risk of bias (including confounding), inconsistency, indirectness, imprecision, and publication bias, and was downgraded when serious or very serious concerns were identified, in accordance with GRADE guidance for prognostic factor studies. A Summary of Findings table was generated to present relative effect estimates and the overall certainty of evidence for each outcome (Supplementary Table S1).

## Results

A total of 573 articles were found in database search: 308 from PubMed and 265 from Cochrane. After the removal of 88 duplicates, 485 records were subjected to title and abstract screening. Of these, 49 articles further underwent full text assessment for eligibility, and 17 studies met the inclusion criteria which were included for the qualitative and quantitative analysis.

### Baseline characteristics

The pooled analysis included a total of 1,068 patients from 17 studies. The mean age of participants in the individual studies ranged from 23.4 ± 4.8 years to 33.0 ± 8.2 years, with the majority of studies reporting mean ages in the mid-to-late twenties. The proportion of male participants varied across the included studies, ranging from as low as 13% in some treatment groups to as high as 78%. Atrophic acne scars, including subtypes such as boxcar, rolling, and ice-pick scars, were the most frequently reported type of acne scarring. Where reported, the severity of acne scars ranged from Goodman and Baron grade 2 to grade 4. A summary of the baseline characteristics of the included studies is presented in Table 1.

**Table 1:** Baseline characteristics of included studies

Study (Year)	Country	Study Design	Sample Size (Total/MN-MNRF-Derma rolling/CO2)	Age (MN vs. CO2)	Male % (MN/MNRF/Derma rolling vs. CO2)	Type of acne scars	Goodman & Baron Grade	Follow Up
Rakput 2021	India	Prospective non-randomized	50/25/25	26.8 ± 7.7 vs. 26.2 ± 6.3	48% vs. 44%	Grade 2–4	2–4	N/A
Hu 2025	China	Split-face	18/15/15	24.7 ± 10.3 vs. 24.7 ± 10.3	60% vs. 60%	Ice-pick, boxcar, rolling	N/A	N/A
Hendel 2023	Denmark	Randomized split-face	15/15/15	N/A	13% vs. 13%	Boxcar, rolling, ice-pick	N/A	N/A
Mallah 2025	Iran	Open randomized	30/15/15	26.3 ± 2.8 vs. 24.9 ± 3.2	33% vs. 60%	Boxcar, rolling, ice-pick	N/A	6 months
Mukhtar 2023	Pakistan	RCT	188/94/94	29.0 ± 5.6 vs. 27.3 ± 5.2	78% vs. 74%	Grade 2–3	2–3	13–16 months
Reddy 2021	India	Clinical trial	30/15/15	N/A	N/A	Ice-pick, mixed	N/A	N/A
Said 2025	Pakistan	Clinical trial	60/30/30	26.8 ± 4.9 vs. 27.4 ± 5.2	43% vs. 47%	Atrophic	N/A	N/A
Laser retrospective	India	Retrospective	32/16/16	N/A	N/A	Atrophic	2–4	N/A
Agrawal 2024	India	Split-face	30/18/12	N/A	N/A	Ice-pick, rolling, boxcar	N/A	> 10 yrs duration
Behrangi 2022	Iran	RCT	90/30/26	33.0 ± 8.2 vs. 29.6 ± 6.3	23% vs. 27%	Atrophic, hypertrophic	N/A	Median 10 months
Obaid 2021	Pakistan	Quasi-experimental	40/17/23	28.2 ± 3.1 vs. 28.2 ± 3.1	N/A	Not specified	N/A	N/A
Pooja 2020	India	RCT	60/40/20	N/A	N/A	Grades 2–4	2–4	Mean 3.5 yrs
Meléndez 2024	Chile	Prospective	9/9/9	30.7 ± 8.6 vs. 30.7 ± 8.6	56% vs. 56%	Boxcar, ice-pick, rolling	N/A	N/A
Thakkar 2025	India	RCT	50/25/25	26.3 ± 3.9 vs. 25.8 ± 4.2	48% vs. 44%	Atrophic	2–3	24 months approx
Monisha 2020	India	RCT	140/70/70	27.4 ± 4.3 vs. 27.0 ± 3.9	44% vs. 41%	Mild–severe, hypertrophic	N/A	N/A
Abel 2020	India	Randomized comparative	200/100/100	N/A	45% vs. 39%	Grade > 3	N/A	N/A
Saoji 2017	India	RCT	50/25/25	23.4 ± 4.8 vs. 23.4 ± 4.8	52% vs. 48%	Grades 2–3	2–3	N/A

### Study characteristics

All 17 included studies were clinical trials published between 2017 and 2025, with the majority (*n* = 12) conducted in India. Other studies originated from Pakistan (*n* = 3), Iran (*n* = 1), China (*n* = 1), Denmark (*n* = 1), and Chile (*n* = 1). The study designs included randomized controlled trials (RCTs), split-face studies, and prospective or retrospective clinical trials. Sample sizes varied considerably, ranging from 9 to 200 participants. The duration of patient follow-up was reported in five studies, ranging from a median of 10 months to over 10 years. Patients were treated with either microneedling (MN), microneedling with radiofrequency (MNRF), dermarolling, or fractional CO₂ laser, with comparisons made between these interventions across the primary and secondary outcomes.

### Quality assessment

A structured risk-of-bias appraisal was performed for all included studies using design-appropriate tools. Overall, the methodological quality of the included evidence was considered high; only a limited number of studies demonstrated concerns in isolated domains.

### Observational studies

**Fig. 3:**
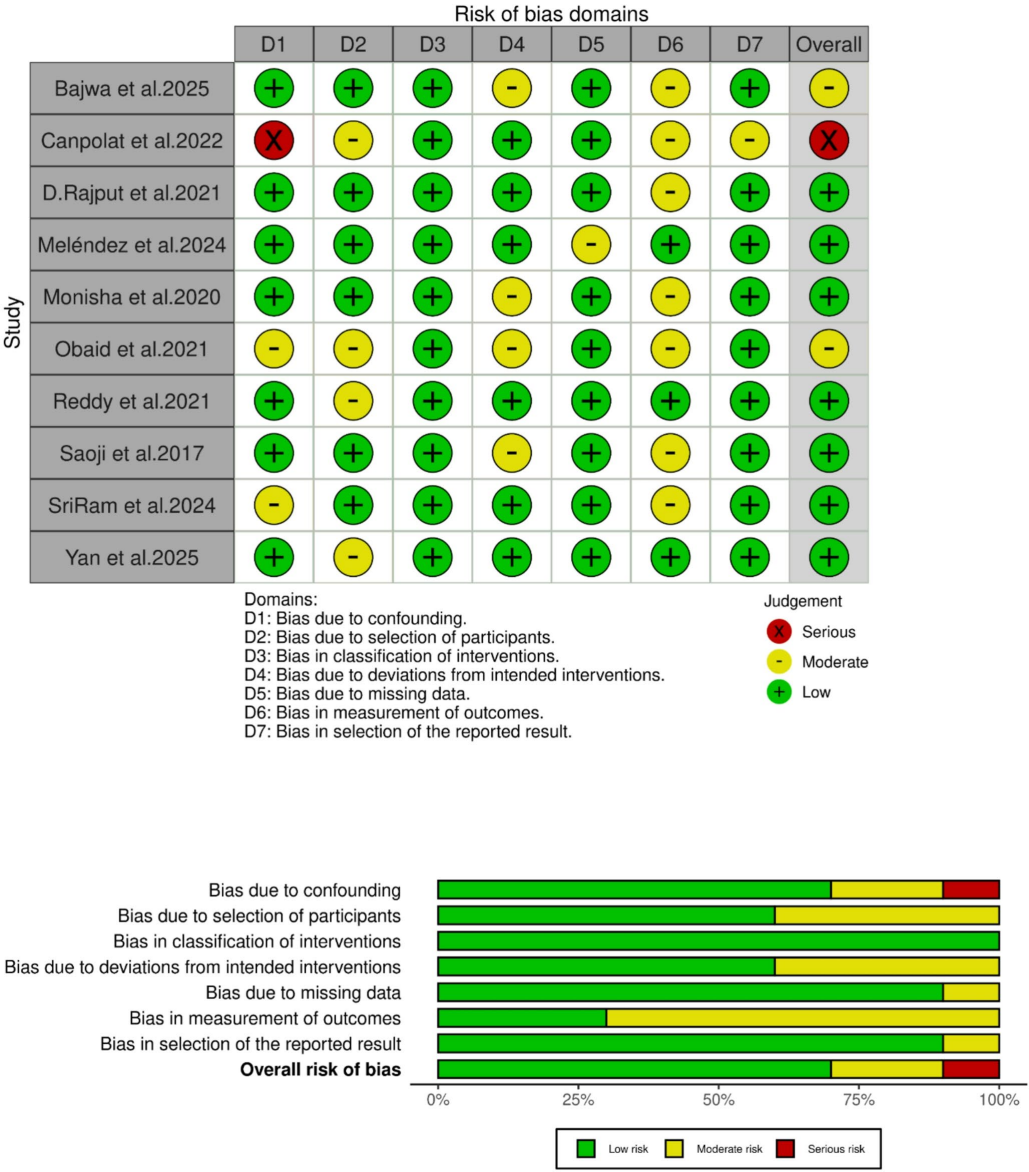

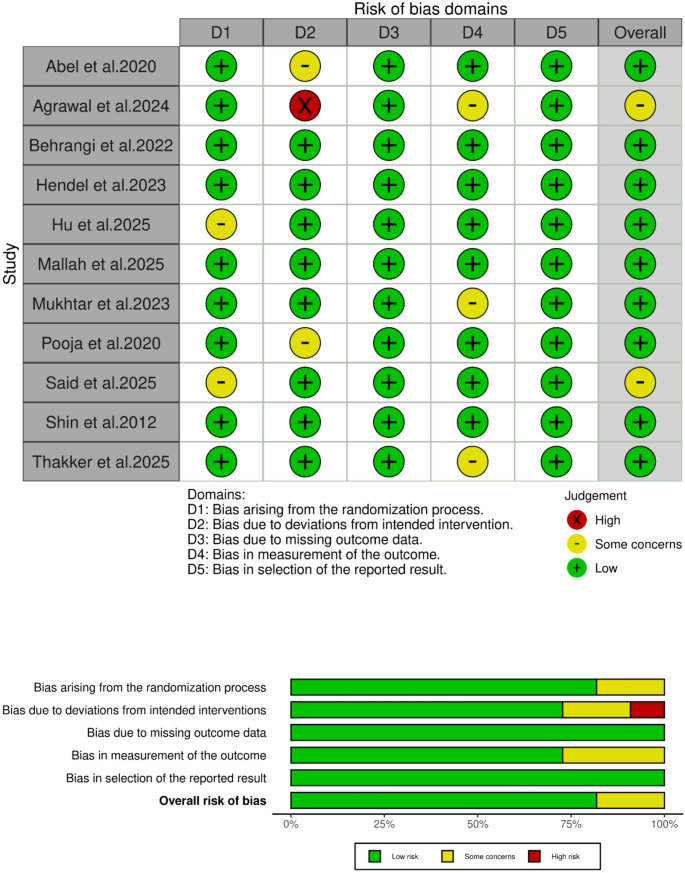
**A** A summary of Cochrane ROBINS-1 assessment. **B** A summary of Cochrane ROB2 assessment

The risk-of-bias assessment for the observational studies was assessed using ROBINS-I across seven domains. Most studies showed a low risk of bias for most domains, showing generally good observational design and reporting. Judgments of low risk were predominant in participant selection, classification of interventions, deviations from intended interventions, completeness of outcome data, and measurement of outcome. Few studies reported isolated concerns. Bajwa et al. (2025) and Agrawal (2024) were considered to have a moderate risk in the domain of confounding (D1), mainly because baseline differences were not fully adjusted for. Melendez (2024) revealed moderate concerns regarding outcome measurement (D6). One of the older studies, Shin et al. 2012, had a serious risk regarding selective reporting (D7) and was therefore also rated as having a serious overall risk of bias. The domain-level summary indicated that more than 85–90% of judgments across observational studies were low risk, although there was the occasional moderate or serious concern confined to specific domains. Not much high bias was found in observations and therefore the observational evidence is reliable. This is presented in Fig. 3A (A summary of Cochrane ROBINS-1 assessment) and Supplementary Table S2 (Robins-1 assessment).

### Randomized controlled trials

We used the RoB-2 framework to perform a risk-of-bias assessment for RCTs across five domains. The majority of the included trials had been evaluated as presenting low risk of bias, particularly pertaining to adherence to intended interventions, handling of outcome data, measurement of outcomes, and selection of reported results. Two studies—Hu et al. (2025) and Behrangi et al. (2022)—were judged to have some concerns regarding randomization (D1), mainly due to a lack of detail on allocation concealment. Thus, these studies were judged to have some overall concerns, whereas the remaining RCTs were all low overall risk. The aggregated summary revealed that over 90% of the domain-specific assessments across the RCTs were of low risk; only small concerns existed in the randomization process. No trial showed high or serious risk in any of the domains. This is presented in Fig. 3B (A summary of Cochrane ROB2 assessment) and Supplementary Table S3 (ROB2 assessment).

### Overall quality interpretation

This reflects that the quality assessment shows the studies included in this meta-analysis are methodologically confident, as the large majority of studies show a low risk of bias. However, a few studies have moderate or serious concerns in some domains. These were not wide and thus are not likely to have a major effect on the validity of the pooled effects. The overall evidence is thus considerately reliable and is of high quality and is in support of confidence in the meta-analytic findings.

### Primary outcomes

#### Mean reduction in acne scar score

**Fig. 4:**
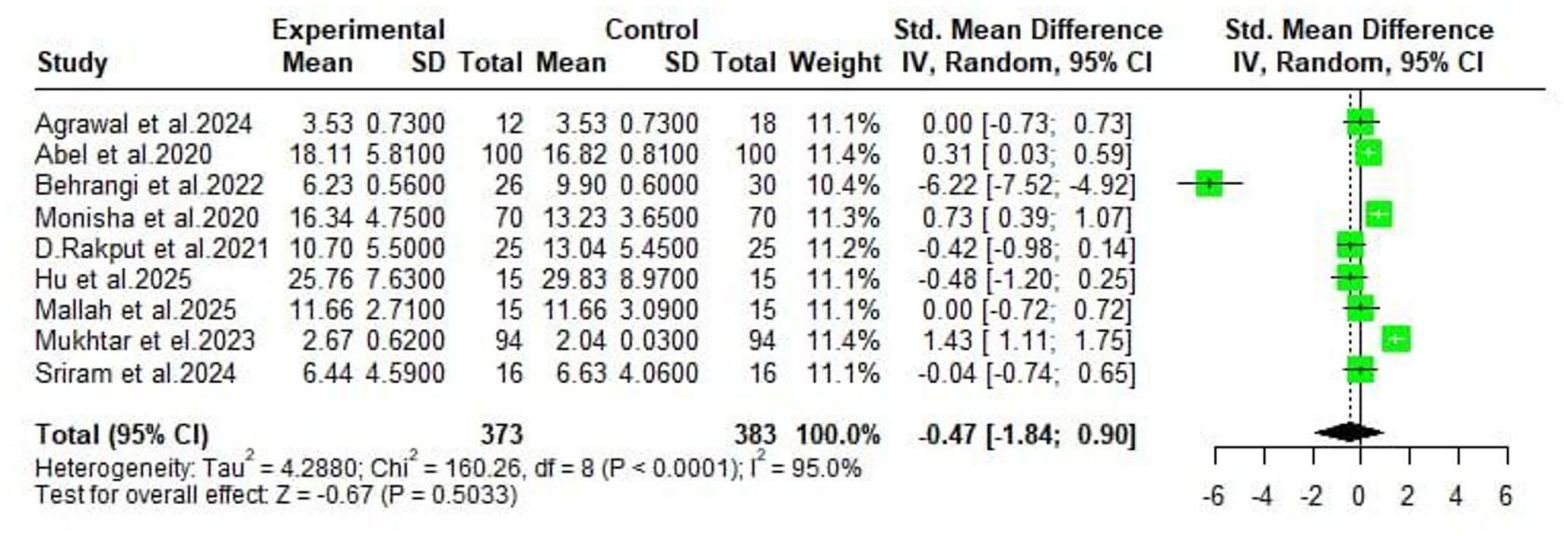

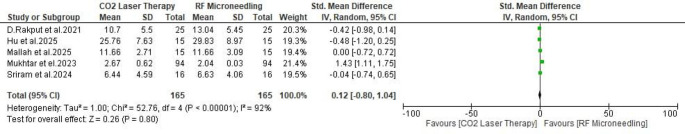

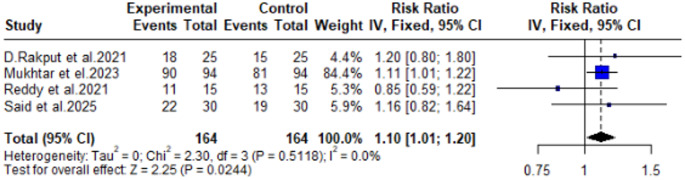
A Forest plot of mean Reduction in Acne Scar Score. B Forest plot of mean Quantitative Scar Reduction C Forest plot of categorical Treatment Success

Four trials mentioned mean reduction in acne scar score, comprising 208 participants treated with fractional CO₂ laser and 218 treated with microneedling. Analysis was done using a random effect model. The pooled results showed no statistically significant difference between fractional CO₂ laser and microneedling: SMD = − 1.08; 95% CI: − 2.48 to 0.32; *p* = 0.13. There was, however, high heterogeneity: Tau² = 1.89; χ² = 102.54, df = 3, *p* < 0.00001; I² = 97%, showing significant variability across different studies results. Figure 4A.

##### Sensitivity analysis

After removing the highly influential study Behrangi et al., 2022 (SMD − 6.22), the pooled result became statistically significant, favoring fractional CO₂ laser therapy (SMD = 0.42; 95% CI: 0.05–0.79; *p* = 0.03), and heterogeneity decreased to a moderate level (I² = 60%).

This suggests that the nonsignificant effect was predominantly driven by outlier study Behrangi et al., 2022. This is shown in Supplementary Figure S1B.

#### Mean quantitative scar reduction

Five trials having 330 participants in total (165 in each group) showed mean quantitative reduction, comparing fractional CO₂ laser therapywith RF microneedling. The pooled results demonstrated, there was no significant difference between fractional CO₂ laser and microneedling: SMD = 0.12; 95% CI: − 0.80 to 1.04; *p* = 0.80. Here also the hetrogenicity was very high: χ² = 52.76, df = 4, *p* < 0.00001; I² = 92%. Figure 4B.

##### Sensitivity analysis

Sensitivity analysis was done and after excluding the influential study of Mukhtar et al., 2023 (SMD = 1.43),results showed a statistically significant effect favoring RF microneedling SMD = − 0.26; 95% CI: − 0.59 to − 0.08; *p* < 0.05, and heterogeneity became 0%.

This concluded that the original pooled estimate was unstable and sensitive to the presence of outlier Mukhtar et al., 2023. This is shown in Supplementary Figure S2A.

#### Categorical treatment success

Four trials having 328 participants, 164 in each group, reported categorical treatment success as an outcome. Results showed, 141 participats in the fractional CO₂ laser group showed improvement while 128 in the RF microneedling group. The pooled risk ratio showed that treatment success was significantly higher for CO₂ laser therapy (RR = 1.10; 95% CI: 1.01–1.20; *p* = 0.02). This is presented in Fig. 4C. For the categorical treatment success the heterogeneity was minute: χ² = 2.30, *p* = 0.51; I² = 0%. So, all the findings were consistent across studies. This suggests a modest advantage of fractional CO₂ laser therapy in contrast to rf microneedling.

### Secondary outcomes

#### Pain score (VAS)

##### CO₂ laser vs. standard microneedling

Two trials having 68 participants, 34 in each group, reported pain scores. The pooled effect showed no significant difference between fractional CO₂ laser and standard microneedling, SMD = − 0.81, 95% CI: − 4.91 to 3.28, *p* = 0.70, with very high heterogeneity making the results non conclusive.

##### CO₂ laser vs. RF microneedling

Two trials involving 60 participants reported pain scores. It was significantly low with fractional CO₂ laser therapy; SMD = − 0.74, 95% CI: − 1.27 to − 0.21,*p* = 0.006.

Here the heterogeneity was I² = 0%, so we have very consistent findings.

**Fig. 5:**
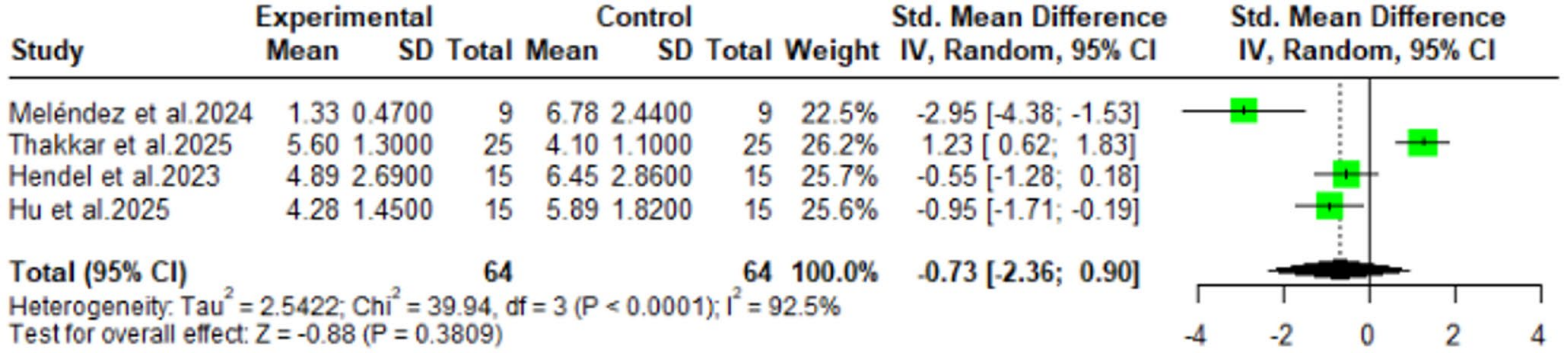

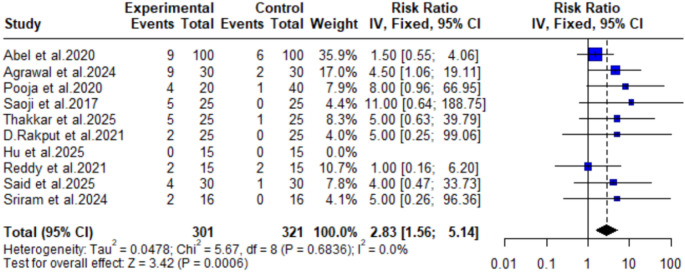

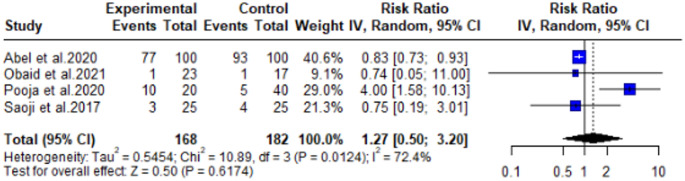

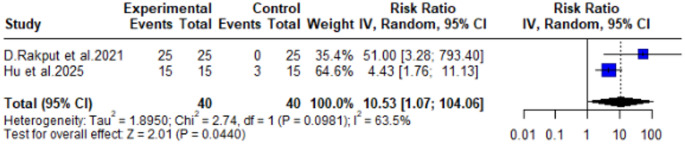

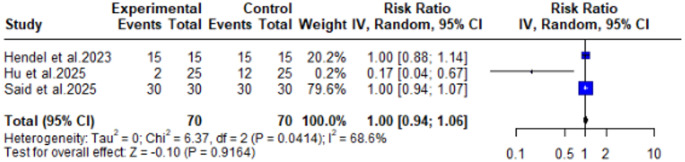
**A** Forest plot of pain Score (VAS). **B** Forest plot of Post-Inflammatory Hyperpigmentation (PIH). **C** Forest plot of post-Procedure Erythema. **D** Forest plot of crusting/Scabbing. **E** Forest plot of edema

This analysis is presented in Fig. 5A.

#### Post-Inflammatory hyperpigmentation (PIH)

##### CO₂ laser vs. standard microneedling

PIH was reported by five studies having a total of 420 participants. Fractional CO₂ laser therapy was associated with a significantly increased risk of PIH, RR 3.04 (95% CI: 1.49–6.21; *p* = 0.002), and heterogeneity is low (I² = 3%).

This shows a significantly higher risk of PIH with CO₂ laser vs. patients undergoing standard microneedling.

##### CO₂ laser vs. RF microneedling

Four trials with 202 participants showed 10 events of PIH in fractional CO₂ laser group and 3 in RF microneedling group. The results however does not show statistical significance: RR = 2.45; 95% CI: 0.77–7.80; *p* = 0.13; I² = 0%.

This analysis is presented in Fig. 5B.

#### Post-procedure erythema

Four trials having 350 participants showed results for erythema. The pooled effect showed no statistical difference between fractional CO₂ laser and microneedling: RR = 1.26; 95% CI: 0.45–3.57; *p* = 0.66, and heterogeneity is also high, I² = 77%. This analysis is presented in Fig. 5C.

##### Sensitivity analysis

Removing the outlier, Pooja et al. 2020, heterogeneity was minimized (I² = 0%) and the pooled result shows co2 laser resduced redness, with an RR of 0.83; 95% CI: 0.73–0.93; *p* = 0.002, thus showing benefit of fractional CO₂ laser therapy. This is shown in Supplementary Figure S5B.

#### Crusting/scabbing

Two studies with 80 participants, 40 in each group, gave results for on crusting/scabbing. Three events occurred in the fractional CO₂ laser group and zero in the RF microneedling group.

Although the relative risk was high (RR = 12.08), the pooled result is not statistically significant (95% CI: 0.60–243.67; *p* = 0.10), and heterogeneity was high (I² = 77%). This analysis is presented in Fig. 5D.

#### Edema

Three trials with 140 participants, reported edema. The pooled effect showed no significant difference between fractional CO₂ laser and RF microneedling, RR = 0.86; 95% CI: 0.54–1.35; *p* = 0.50, also the heterogeneity was very high in results, I² = 96%. This analysis is presented in Fig. 5E.

##### Sensitivity analysis

Sensitivity analysis was done by excluding the outlier, Hu et al., 2025, the result became stable but still remained not significant (RR = 1.00; 95% CI: 0.94–1.06; *p* = 1.00; I² = 0%). This is shown in Supplementary Figure S6B.

## Discussion

In this meta-analysis, fractional CO₂ laser and microneedling treatments provided comparable overall outcomes for the treatment of atrophic acne scars as there was no statistically significant difference between them in regard to mean scar reduction. Fractional CO₂ laser was associated with slightly higher odds of categorical treatment success, but higher risk of post-inflammatory hyperpigmentation. We also observed that radiofrequency (RF) microneedling tended to be more painful intra-procedurally than fractional CO₂ laser, though other sequelae (erythema, edema, crusting) did not consistently differ between modalities.

The lack of significant difference between the two modalities in terms of mean scar improvement suggests that both ablative fractional lasers and microneedling (including RF microneedling) lead to two different mechanisms for dermal remodeling but both have an equal clinical effect. Fractional CO₂ laser is the gold standard [28], ablating epidermal and dermal columns to induce production of new collagen [29], while microneedling generates controlled micro-injuries and with RF delivers heat to the dermis without harming the epidermis [30]. Both pathways culminate in similar advancements for quality of scar (texture and depth) and permit those unsuitable for laser — e.g. darker skin types or contra-indications — to still demonstrate meaningful change [29]. The slight advantage in categorical success with lasers may reflect more aggressive remodeling, but the benefit is modest, reinforcing the therapeutic equivalence and accessibility of microneedling-based options [19].

Our efficacy findings largely accord with the existing literature on acne scar treatments. Fractional RF technology achieved scar improvement that was equivalent to that of fractionated ablative lasers [22], which is exactly what we observed overall. Hendel et al. also observed similar texture improvement of fractional CO₂ laser and RF microneedling in a split-face comparative study [31], and Rajput et al. found a similar decrease in scar severity (~ 60–63%) and proportion of patients achieving > 50% reduction [32]. A laser superiority is supported by some studies; for example, an Indian split-face trial reported superior response to fractional CO₂ laser with more hyperpigmentation seen [19]. Taken together, the evidence suggests that while lasers may confer marginal additional scar improvement in certain contexts, microneedling (especially RF-enhanced) can achieve equivalent clinical benefits in most patients when considering the balance of outcomes.

A key safety finding is the increased risk of PIH with fractional CO₂ laser as has been reported in darker skin types [19]. RF microneedling largely spares the epidermis to cause less PIH and has no significant difference with laser [22]. Previous studies also recorded minimal PIH in RF-treated Fitzpatrick III–V patients [32], consistent with modality choice by phototype [19].

Procedural pain was also remarkable difference.Although ablative lasers are expected to be more painful, our analysis found RF-microneedling produced equal or greater pain than fractional CO₂ lasers.In a large propensity-score cohort, the mean pain scores were significantly higher for FRF vs. fractional lasers (5.65 ± 1.74 and 4.14 ± 1.83; *P* < 0.001, respectively) [22].However, a split-face pilot study found higher pain on the AFCO₂ side (mean of 8 versus 3 by VAS) with longer duration of 16 compared to 4 h.While one MNRF preliminary study reported mean scores of pain at only 1.5/10 and no adverse effect [33, 34].Such variation is probably due to differences in anesthetic administration, because robust multimodal analgesia is often used with fractional CO₂ laser [35–37], and microneedling uses topical lidocaine [34].Both treatments remained well tolerated with no discontinuations [22]. Recovery favored microneedling, with AFCO₂ showing markedly longer erythema, edema, crusting, and PIH [32, 33], consistent with clinical experience [37]. These differences in tolerability and recovery are critical considerations when counseling patients on their treatment options.

The heterogeneity across trials comes likely from protocols’ and patients’ selection diversity. The outlier study excluded in sensitivity analysis was microneedling plus platelet-rich plasma which universally improves outcomes and obscures potential benefit from a laser side, demonstrating that adjuvants can alter results. Scar morphology, treatment intensity (higher-fluence lasers, deeper microneedling), and session number (one to four) also influenced improvement, reflecting treatment “dose”. Methodological factors—variable success thresholds, lack of blinding, lower laser settings in Asian populations to limit PIH risk, and differences in technique—further shaped results, proving that outcomes were context-dependent. In clinical practice, such light-skinned individuals with significant scarring may benefit from lasers, although microneedling affords the similar improvement with decreased risk [22, 32].

### Limitations

Notwithstanding the merits of this meta-analysis, many limitations must be recognized. Trials were few in number and had low sample sizes, limiting statistical power and confidence. A high heterogeneity indicated discrepancy of study designs, patient populations, and outcome reporting; various scar scales and improvement criteria led to standardized mean differences that may not necessarily reflect the magnitude at clinical level. Interventions also varied: “microneedling” included standard and RF techniques, and laser protocols differed in devices and settings, which may confound pooled estimates.

Patient populations tended to be restricted, and the majority were from Asia or Middle East, thus possible generalization to other race and skin type was limited. Postoperative follow-up was short-term, so the durability long-term and late complications are unknown. Publication bias could not be evaluated, and results regarding outcomes such as quality of life, satisfaction, cost effectiveness were inadequately reported. These limitations need to be considered, and further larger diverse randomized trials with standardized outcomes measures, longer follow-up patient-reported outcomes combination-therapy evaluation PIH-mitigation strategies along with the assessment of cost-effectiveness are needed.

## Conclusion

Fractional CO₂ laser and microneedling are equally effective for atrophic acne scars. The CO₂ laser leads to slightly more improvement but is associated with increased risk of PIH and downtime. In particular, RF-based microneedling is able to induce parallel remodeling with a better safety profile in skin from patients that are pigment-prone and faster recovery. Neither is therefore inherently “better,” but rather that it should depend on patient variables such as skin type, types of scars, and willingness to have downtime or possible complications. Each of these approaches represent useful techniques for the treatment of acne scars, and our findings emphasize the need to further tailor therapies in order to maximize benefit without greatly increasing risk.

## Supplementary information

Below is the link to the electronic supplementary material.

Supplementary Material 1

## Data Availability

All data were obtained from publicly available sources. Relevant data supporting the findings of this study was included in the manuscript and supplementary material. Additional details can be shared upon reasonable request to the corresponding author.

## Abbreviations

AFCO₂: Ablative Fractional Carbon Dioxide
CIs: Confidence Intervals
CO₂: Carbon Dioxide
CRD: Centre for Reviews and Dissemination (PROSPERO Record ID prefix)
D1: D7–Risk of Bias Domains (ROBINS–I)
ECCA: Echelle d’Evaluation Clinique des Cicatrices d’Acné
FRF: Fractional Radiofrequency
GRADE: Grading of Recommendations Assessment, Development and Evaluation
MD: Mean Difference
MN: Microneedling
MNRF: Microneedling Radiofrequency
PIH: Post–Inflammatory Hyperpigmentation
PICO: Population, Intervention, Comparison, Outcome
PRF: Pulsed Radiofrequency
PROSPERO: Prospective Register of Systematic Reviews
RCT: Randomized Controlled Trial
RF: Radiofrequency
ROB: 2–Risk of Bias 2 Tool
ROBINS: I–Risk Of Bias In Non–randomized Studies of Interventions
RR: Risk Ratio
SMD: Standardized Mean Difference
VAS: Visual Analogue Scale
